# Mental health professionals and telehealth in a rural setting: a cross sectional survey

**DOI:** 10.1186/s12913-023-09083-6

**Published:** 2023-02-27

**Authors:** David Nelson, Maxime Inghels, Amanda Kenny, Steve Skinner, Tracy McCranor, Stephen Wyatt, Jaspreet Phull, Agnes Nanyonjo, Ojali Yusuff, Mark Gussy

**Affiliations:** 1 Lincoln International Institute for Rural Health (LIIRH), University of Lincoln, Lincoln, UK; 2 Centre Population et Développement (UMR 196 Paris Descartes – IRD), SageSud (ERL INSERM 1244), Institut de Recherche pour le Développement, Paris, France; 3 La Trobe Rural Health School, La Trobe University, Bendigo, Australia; 4 Lincolnshire Partnership NHS Foundation Trust, Lincoln, UK; 5 Sherwood Forest Hospitals NHS Foundation Trust, Nottingham, UK

**Keywords:** Telehealth, Telemedicine, Psychological therapies, COVID-19 pandemic, Rural health, IAPT, United Kingdom

## Abstract

**Background:**

Telehealth usage has been promoted in all settings but has been identified as a panacea to issues of access and equity in the rural context. However, uptake and widespread integration of telehealth across all parts of the health system has been slow, with a myriad of barriers documented, including in rural settings. The crisis of the COVID-19 pandemic, saw barriers rapidly overturned with the unprecedented and exponential rise in telehealth usage. The uniqueness of the crisis forced telehealth adoption, but as the urgency stabilises, pandemic learnings must be captured, utilised, and built upon in a post-pandemic world. The aim of this study was to document staff experiences and perceptions of delivering rural psychological therapies via telehealth during the pandemic and to capture learnings for future rural telehealth delivery.

**Methods:**

An online cross-sectional survey that explored mental health professional’s experiences, use, and perceptions of telehealth before and after pandemic-enforced changes to service delivery.

**Results:**

Sixty-two respondents completed the questionnaire (response rate 68%). Both the delivery of telehealth via telephone and online video conferencing significantly increased during the pandemic (66% vs 98%, *p* < .001 for telephone and 10% vs 89%, *p* < 0.001 for online video). Respondents indicated that client’s access to services and attendance had improved with telehealth use but their attention and focus during sessions and non-verbal communication had been negatively affected. The challenges for older adults, people with learning and sensory disabilities, and residents in remote areas with poorer mobile/internet connectivity were identified. Despite these challenges, none of the respondents indicated a preference to return to fully face-to-face service delivery with most (86%) preferring to deliver psychological therapies fully or mostly via telehealth.

**Conclusions:**

This study addresses three major gaps in knowledge: the experience of delivering local telehealth solutions to address rural mental health needs, the provision of strong rural-specific telehealth recommendations, and the dearth of rural research emanating from the United Kingdom. As the world settles into a living with COVID-19 era, the uniqueness of the rural telehealth context may be forgotten as urban myopia continues to dominate telehealth policy and uptake. It is critical that rural resourcing and digital connectivity are addressed.

## Background

This study documents staff experiences and perceptions of delivering rural psychological therapies via telehealth and captures critical learnings for future rural telehealth delivery. Three major gaps in knowledge are addressed: the experience of delivering telehealth solutions to meet rural mental health needs, the provision of strong rural specific telehealth recommendations, and the dearth of rural research emanating from the United Kingdom (UK).

For decades, telehealth has been central in the digital strategies of major countries to support long-distance healthcare, education, public health, and administration [1–4]. In the rural context, telehealth has been extensively promoted to ensure safe, effective, and equitable service delivery to people impacted by distance and isolation [5–16].

In the pre-pandemic period, urgent calls were made for radical approaches to technological use in healthcare [1–3, 17].Authors of systematic reviews on telehealth had demonstrated client satisfaction, including improved outcomes (varied definitions), cost savings, better communication and ease of use, and reductions in travel time [18]. There were pockets of excellence in telehealth delivery, however, uptake and widespread integration was slow [1–3, 17]. Barriers to uptake included a lack of infrastructure, fractured and complex health systems, and unwieldy funding models/jurisdictional boundaries. Challenging technology and commercial arrangements, risk adversity, limited resources and increased costs, issues with public trust and data security, lack of digital skills amongst workers at all levels and resistance to education, and lack of political and organisational leadership to drive change have all been documented [1–3, 17]. Specific rural barriers included lack of digital connectivity, coordination and turnover of senior management, lower levels of health literacy, lack of resourcing at the client end, challenges in disciplines where touch is a requirement, concerns about security, inadequate staffing, lack of equipment and other resources, inflexible billing arrangements, complex referral patterns, and lack of local control [5–16, 19]. In 2018, in the UK, harsh criticism was directed at an increasing gap between technological potential and usage [1].

### The impact of the COVID-19 pandemic

In 2020, the COVID-19 pandemic saw an unprecedented and rapid overturning of many barriers and major acceleration of telehealth usage [3, 4, 17, 20–22]. In May 2020, Dr. Sacha Bhatia stated, ‘… the COVID pandemic was the match that lit the fire around this revolution in virtual care’. [4] The US Congress rapidly overturned telehealth billing and reimbursement restrictions, enabled telehealth from people’s homes (including physicians), and expanded approved platforms [3]. Similar changes were made in Australia [21, 22], Canada [4, 17, 19], and the UK [23].

Rates of pandemic related anxiety, depression, post-traumatic stress disorder, psychological distress, and stress across the general population [24] [25, 26], saw increased mental health service demand. This demand and reductions in service delivery due to COVID-19 controls, forced service providers to pivot rapidly to telehealth to maintain care delivery.

Key articles have emerged on telehealth for rural mental health service delivery during the pandemic [27, 28]. In rural Pennsylvania, Svistova and colleagues [27] explored mental health provider’s experiences of using telehealth with youth and older populations. Positives included service continuation, greater parental involvement, decreased no-show rates, and easing of transport difficulties. They did, however, note challenges in digital access and recommended hybrid models of service delivery [27]. In a study investigating telehealth usage among people with mental illness in rural Louisiana, Sizer et al. [28] argued that the digital divide required elimination, especially for older people, those with lower levels of education, and those with serious and enduring mental illness. In Virginia, US, telehealth usage during the pandemic was explored for people with adjustment disorders, anxiety, and depression [29]. It was hypothesised that lower uptake of rural telehealth may have been the result of fewer resources to manage demand surge. In Australia, Chatterton et al. [30] demonstrated marked increases in mental health telehealth usage because of the pandemic. Limitations associated with small sample sizes impact their findings, but lack of digital infrastructure, lack of user-friendly platforms, and privacy concerns were reported [30].

Caffery et al. [31] (p.544) argue that the COVID-19 pandemic uncovered ‘a myopia we term urban paternalism in understanding and delivering rural health’. This was described as policy and practice driven from an urban stance. The uniqueness of the pandemic and associated lockdown measures resulted in urban policymakers and urban dwellers experiencing isolation and the inability to access health services. These issues have been documented in the rural context for decades.

While much is documented on rural health in countries with large geographic mass and dispersed populations there is a void in rural research in the UK. It is often forgotten that 85% of the landmass in the UK is rural and is home to more than 10 million people. While parts of rural UK represent the bucolic idyll that many imagine, deprivation, poor outcomes, major inequalities, and unmet needs are often hidden due to a lack of fined-grained data at the county level. Within relatively small geographic areas, life expectancy can vary by 10 years [32]. There are similar issues shared with other rural areas including aging populations, limited access to health and social care, inequalities associated with travel and transport, and generational poverty exacerbated by inadequate housing and escalating fuel costs [32]. Social isolation and loneliness are major features [33]. There are major issues with rural digital exclusion which exacerbates access and social isolation [34].

In 2019, the UK Government released key policy on rural research priorities. They reinforced the diversity in rural UK and need for locally developed strategies, digital connectivity, and technology [34]. The study described in this paper is a report of a major local strategy to address both mental health and rural isolation so is important in the genesis of embryonic UK rural research.

### Study aim

The aim of this study was to document staff experiences and perceptions of delivering rural psychological therapies via telehealth during the pandemic and to capture learnings for future rural telehealth delivery. The research question was: what were mental health professionals’ experiences, use and perceptions of telehealth before and after pandemic enforced changes to service delivery?

### Setting

The setting for this study was the UK county of Lincolnshire. The predominantly rural county has a mixture of affluence and deprivation [35]. Within the county, high rates of smoking, alcohol and drug use, and poor physical and mental health are evident in coastal and deprived communities. Rural, seasonal and coastal populations provide significant challenges to traditional modes of service delivery often based on urban modelling [36].

The Improving Access to Psychological Therapies (IAPT) programme has been implemented nationally in the UK as an evidence-based approach to delivering psychological therapies for depression and anxiety disorders in line with the stepped-care clinical guidelines issued by the National Institute for Health and Care Excellence (NICE) [37, 38]. For people accessing IAPT with anxiety disorders, cognitive behavioural therapy (CBT) is recommended [38]. For those with depression, a wider range of treatments are recommended (CBT, counselling, couples therapy, interpersonal therapy, and psychodynamic therapy) [38]. In England, there are now over 200 IAPT services which render it the largest publicly funded and systematic implementation of evidence-based psychological care in the world [39]. It now serves as a model for similar systems in countries such as Australia, Canada, Norway, and Japan [40–43].

Steps2change (Lincolnshire Partnership NHS Foundation Trust) is the Improving Access to Psychological Therapies (IAPT) service for the county of Lincolnshire in the East Midlands of England. The service is delivered using a nationally developed stepped-care approach offering the least intrusive intervention first and monitoring people’s progress in outcome-focused supervision. Before the COVID-19 pandemic, the service operated mainly from nine sites offering a mixture of one-to-one and group face-to-face appointments, telephone-guided self-help, and to a lesser extent internet-enabled therapy such as computerised cognitive behavioural therapy (cCBT).

## Methods

### Design

An online study-specific cross-sectional questionnaire was administered to staff within the IAPT service at Lincolnshire Partnership NHS Foundation Trust. The survey was designed using Qualtrics software [44].

### Participants

All ninety-one IAPT service staff were invited to participate by email from the IAPT Clinical Lead on behalf of the research team. The IAPT service staff consists of an interdisciplinary team of cognitive behavioural therapists, counsellors, employment advisors, interpersonal therapists, and psychological well-being practitioners. Staff provide a range of evidence based talking therapies and psychological treatments for people with depression, anxiety, panic attacks, post-trauma reaction, phobias and obsessive-compulsive disorders. Those with management/administration-only roles were excluded as they did not have direct experience in delivering telehealth services to people. Trainees and staff who had been recently employed were also excluded as they did not have sufficient experience in delivering IAPT pre and post COVID-19. To maximise responses two reminder emails were sent from the Clinical Lead for the IAPT service to potential participants.

### Questionnaire

The questionnaire was developed from the published literature [45–50] by the core research team in collaboration with IAPT service provider staff. The questionnaire was piloted with four members of the IAPT team to ensure readability and face validity before distributing to all eligible staff. Those who took part in the pilot suggested that free text questions should be added to specific survey items to allow the participants to elaborate on some of their choices. This amendment was made before distribution.

The final questionnaire included demographic questions and items that asked about respondent’s use of telehealth before and after COVID-19 related service changes, their own experiences of telehealth delivery, their perceptions of the level of their client’s satisfaction and perceived impact on the quality and quantity of service provided. There were twenty-four questions in total. Where participants were asked to compare telehealth services with service delivery prior to the Covid-19 pandemic a Likert scale with the following categories was utilised (1) much worse (2) somewhat worse (3) about the same (4) somewhat better and (5) much better. The force response option in Qualtrics was used to ensure that participants did not overlook or miss any of the questions that were vital to the analysis. There was no missing data.

Data were collected between April-June 2021. During this time there were significant restrictions on non-essential social contact imposed by the UK government.

### Analysis

Descriptive statistics (frequencies and percentages) were used to describe and summarise the data. Fisher’s Exact Tests [51] were used to test for significant differences between telehealth use prior to and since the pandemic, as well as changes in perceived skill level before and after the pandemic onset. Free text responses were reported to allow further context to participant’s responses. Data were analysed using R software version 4.1.1 [52].

## Results

### Sample demographics

A total of 62 respondents completed the questionnaire resulting in a response rate of 68%. Whilst there were responses across the different occupations that make up IAPT, cognitive behavioural therapists and psychological and wellbeing practitioners were the highest responders. The responses were a good representation of the different occupations that make up the IAPT service at the time of data collection. Full respondent characteristics are displayed in Table 1.

**Table 1:** Characteristics of respondents and non-respondents

	*Total n = 62* *n* (%)	
Variable		
*Age*		
18-24	3 (4.8)	
25-34	25 (40.3)	
35-44	9 (13.4)	
45-54	10 (14.5)	
55-64	14 (22.6)	
65-74	1 (1.6)	
*Gender*		
Female	50 (80.6)	
Male	12 (19.4)	*Total n = 91*
		*No. eligible to take part n (%)*
*Occupation*		
Cognitive Behavioural Therapist	22 (35.5)	35 (38.4)
Counsellor	8 (12.9)	14 (15.4)
Employment Advisor	9 (14.5)	11 (12.1)
Interpersonal Therapist	3 (4.8)	9 (9.9)
Psychological Wellbeing Practitioner	20 (32.3)	22 (24.2)

*Note: Total data were not available for age and gender only occupation. This number excludes trainee staff*

### Pre and post COVID-19 use of telehealth and reported skill level

Table 2 presents findings about telehealth provision and self-rated skill levels prior to and since COVID-19. Before the pandemic, two-thirds (66%) of staff had delivered services via the telephone but only 10% reported the use of online video conferencing for service delivery before the pandemic. The reported delivery of telehealth via both telephone and online video conferencing had significantly increased since the pandemic with 98% reporting the use of telephone and 89% video conferencing. Additionally, participant’s self-rated skill levels delivering telehealth via the telephone and using video conferencing had significantly improved compared to before the pandemic. Despite this, 58% reported no formal training for using telehealth at the time of data collection (Table 3).

**Table 2:** Telehealth provision, and self-rated skill level within IAPT prior to and since COVID-19 (March 2020)

	Prior to COVID-19	Since COVID-19	
	*Total n = 62* *n* (%)		*p* value*
Telephone			
*Provision of care using telephone consultation*			<.001
Yes	41 (66.1)	61 (98.4)	
No	21 (33.9)	1 (1.6)	
*Skill level using telephone consultation*			<.001
Very good	16 (25.8)	32 (51.6)	
Good	18 (29.0)	25 (40.3)	
OK, but not brilliant	24 (38.7)	5 (8.1)	
Not at all good	4 (6.5)	0 (0.0)	
Online video conferencing			
*Provision of care using online video conferencing*			<.001
Yes	6 (9.7)	55 (88.7)	
No	56 (90.3)	7 (11.3)	
*Skill level using online video conferencing*			<.001
Very good	4 (6.5)	18 (29.0)	
Good	11 (17.7)	33 (53.2)	
OK, but not brilliant	21 (33.9)	8 (12.9)	
Not at all good	26 (41.9)	3 (4.8)	

*Fisher’s Exact Test was used to assess for significance between telehealth use and skill level prior to and since COVID-19 (from March 2020)

**Table 3:** IAPT Practitioner Experiences of Telehealth since COVID-19

	*Total n = 62* *n* (%)	*Example free text responses*
*Perceived patient satisfaction with telehealth compared to face-to-face*		
Higher when compared to F2F	14 (22.6)	“More flexibility in times and being able to offer appointments that suit. No travel.”
Similar levels of satisfaction compared to F2F	40 (64.5)	
Lower levels of satisfaction compared to F2F	8 (12.9)	“Clients still hold a belief that face-to-face treatment will be better. It can be hard to overcome this thinking.”
*Practitioner satisfaction with telehealth since Covid-19*		
Very satisfied	35 (56.5)	
Satisfied	22 (35.5)	
Neither satisfied nor unsatisfied	4 (6.5)	
Unsatisfied	1 (1.6)	
*Preferred form of service delivery*		
Fully Telehealth	20 (32.3)	
Mostly Telehealth with some F2F	33 (53.2)	
Mostly face-to-face with some Telehealth	9 (14.5)	
Fully F2F	0 (0.0)	
*Thinks diary is easier to manage with telehealth*		
Yes, it is easier when compared to F2F	33 (53.2)	
It is about the same effort compared to F2F	27 (43.5)	
No, it is not as easy compared to F2F	2 (3.2)	“Admin is taking longer.”
*Thinks telehealth changes the way confidentiality is maintained*		
Yes, it makes it more difficult	13 (21.0)	“When the individual doesn’t have privacy at home it can be difficult.”
No, it makes little difference	45 (72.6)	
Yes, it makes it easier	4 (6.5)	“Patients do not have to sit in the waiting room where they may know others.”
*Aware of groups who have difficulty engaging with Telehealth*		
Yes	40 (64.5)	“People who live in remote areas who don’t have good internet or phone signal.”
No	22 (32.5)	
*Ever had telehealth related training since COVID-19*		
Yes	26 (41.9)	“Webinars and written guidance.”
No	36 (58.1)	“We have some CPD on this, but no specific training by the Trust I don’t think.”
*Ever had a technology/ICT issue with Telehealth since COVID-19*		
Yes	48 (77.4)	“Poor connection, either on telephone or on-line.”
No	14 (22.6)	
*Thinks that telehealth requires different skills compared to F2F*		
Yes	48 (77.4)	“I sometimes find it harder to close down inappropriate communications and interject when necessary while being unable to use visual techniques.”
No	9 (14.5)	
Not sure	5 (8.1)	

*Note: Free text responses were not available for all questions*

### Experiences and perceptions of telehealth service delivery

**Fig. 1:**
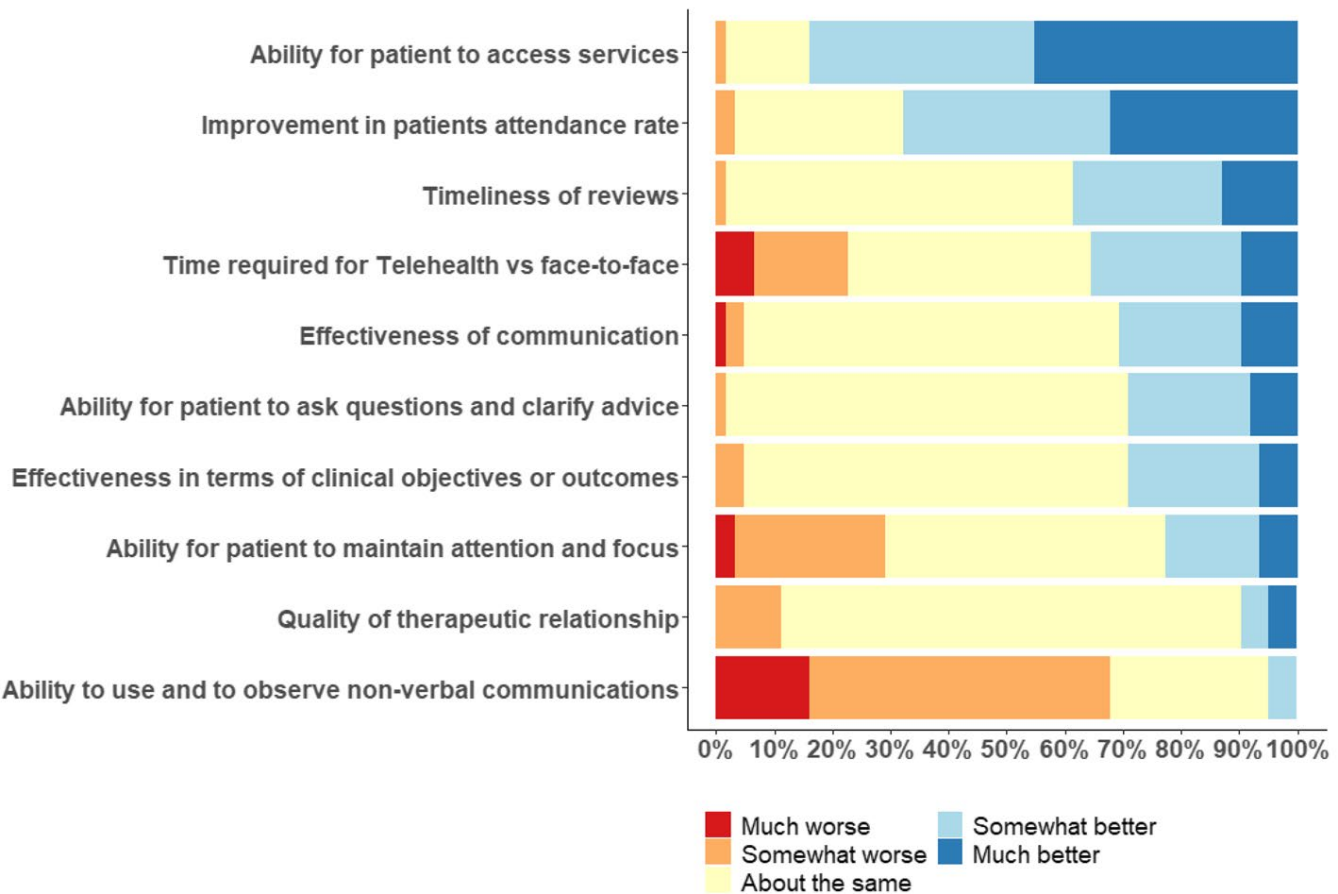
Comparing Telehealth services with services you provided prior to COVID-19 and the move to Telehealth (March 2020), how would you rate the following

Respondents were asked to compare telehealth services with services provided before COVID-19 and the move to telehealth in ten key areas (Fig. 1). Positive changes were reported in client’s access to services as well as attendance rates for telehealth consultations. More negative perceived impacts were the client’s ability to use and observe non-verbal communication as well as to maintain attention and focus. Mixed findings are seen in the perception of time required for each approach to service.

Respondents largely believed client’s had similar or higher levels of satisfaction with telehealth services when compared to face-to-face (Table 3). Most practitioners were themselves satisfied or very satisfied with the delivery of IAPT services via telehealth and none wanted IAPT to return to a fully face-to-face service. There was a strong preference for future services to be mostly or fully online (53 and 32% respectively). Many respondents (58%) indicated that they had not had any formal training in telehealth approaches and/or technologies. This was despite a high level of agreement (77%) that telehealth service delivery required different skills to traditional in-person consultations. When asked about concerns maintaining patient confidentiality when using telehealth approaches most (73%) indicated little difference when compared to pre-COVID practices, although one-fifth reported it as more difficult.

Respondents indicated that there might be groups of people who could have difficulty engaging with telehealth and digital services. These were largely older adults, people with learning and sensory disabilities, as well as those in remote areas prone to poor phone signal and internet connectivity.

## Discussion

The aim of the study was to document staff experiences and perceptions of delivering rural psychological therapies via telehealth during the pandemic and to capture learnings for future rural telehealth delivery. Three major gaps in knowledge are addressed.

The dearth of robust research on rural service delivery, telehealth, and other technological solutions has been documented by the UK Government who have called for research to inform rural policy development and implementation [34]. The Government highlights the need to ‘draw on specific local development strategies and their effectiveness in promoting inclusive growth and welfare.’ [34] Steps2change (Lincolnshire Partnership NHS Trust), the Improving Access to Psychological Therapies (IAPT) service for the county of Lincolnshire in the East Midlands of England, provides an ideal case to illustrate local strategies.

In the pre-pandemic period, the promotion of telehealth, almost as a panacea to address rural geographic distance and health service inequities, did not gain enough traction for widespread, large-scale adoption [1–3, 17]. Much of the existing literature over the last decades has been about chronicling challenges of why telehealth was difficult to implement [1–3, 17]. The COVID-19 pandemic resulted in a seismic paradigm shift as urban dwellers suddenly experienced what rural people had reported for decades: isolation and inability to easily access healthcare [31]. Without diminishing the impact of the pandemic globally, some argue that the rapid overturning of barriers and unprecedented acceleration of telehealth usage [3, 4, 17, 20–22] was because of urban paternalism [31] and urban policymakers who had their myopic blinders about isolation and inequity rapidly blown off. As the world settles into living with COVID-19, there is a major risk that urban myopia will continue to dominate telehealth expansion and the uniqueness of the rural context will be forgotten, resulting in continued inequities across the rural/urban divide.

Our results offer valuable insight into the experiences and perceptions of mental health practitioners using telehealth to deliver psychological therapies during the COVID-19 pandemic. Practitioners believed telehealth approaches had greatly improved patients’ access to IAPT services. Telehealth addressed flexibility around people’s schedules, reduced travel and opportunity costs, and reduced risks of the stigma associated with physically attending healthcare premises for mental health treatment. None of the participants in the study wanted to return to a completely face-to-face model of delivery.

The positives of telehealth identified by practitioners in this study have been identified in studies in the UK, US, Canada and Australia [53–55]. In rural US, Svistova and colleagues [27] recommended hybrid models of service delivery rather than a return to all face-to-face. Additionally, these positives align with systematic reviews on the perspectives of clients, who see improved access and reductions in both the cost and time commitment major benefits of telehealth [18].

The lack of digital infrastructure identified in this study has consistently been reported internationally across a multitude of health systems. In 2018 in the United Kingdom [1], building the best technology into health systems and ensuring that digital systems and people’s needs aligned were identified as critical. It took a global pandemic to expedite action despite decades of calls to improve digital connectivity and ensure the building blocks of the right digital architecture are in place. Urban policymakers and urban dwellers would not tolerate the rural digital access and connectivity issues that have been well-documented for decades [31]. The UK Government is not unique in stating that inferior digital infrastructure is beyond acceptable [2, 3, 19], but these comments are about all areas of the NHS, not just rural. As the crisis of the pandemic diminishes there is a major risk that technology developments will be centred on large urban centres and rural areas will continue to experience digital connectivity issues that are poorer than some low-income countries. Major, targeted investment must be made in rural areas. If personal connectivity cannot be guaranteed, then investment should be made by government to work with technology developers to identify and implement practical solutions to address issues such as sub-standard bandwidth. As noted in this study, it is unacceptable that major sections of the community cannot even achieve a consistent internet connection.

This study demonstrated major gaps in education to ensure that health professionals had the knowledge and skill to deliver outstanding telehealth services. Major investment in the education of all health professionals in telehealth service delivery, no matter the context, has been identified as critical. New roles that span the interface between clinical and technical staff in supporting staff and clients and providing detailed analytics will be needed [56] However, at a fundamental level, rural health professionals face major barriers in accessing education targeted to their needs and locality. There is a dearth of literature on telehealth education for rural health professionals. In a recent scoping review on health professional education in isolated settings, Reeve et al. [57] reported on 40 studies. Digital and technological education was not identified in any study [57]. Massive Open Online Courses (MOOCs) have been proposed as one solution to digital education [56], however, the delivery of urban-centric training programs by online means to outpost rural settings is not without its challenges. Local high quality-education programs must be implemented to ensure high-quality outcomes for both staff and clients. There should be a focus on co-designed educational programs by health professionals and clients that align with their need, and shared learning of staff and clients should be a priority.

In this study, respondents identified groups who might have difficulty engaging with telehealth and digital services. This needs to be flipped. It is not the groups that have difficulty engaging, it is that telehealth and digital services are hard to engage with. Studies that show the most promising telehealth outcomes are largely with well-educated populations [28]. Challenges that older adults, people with mental illness, and people with learning and sensory disabilities face in accessing telehealth documented in this study have been reported in rural US [27, 28]. A simple solution would be to suggest that face-to-face service delivery is better for groups that already face deprivation due to their location [28]. However, studies show high acceptability of telehealth with populations that are well supported [18] so why should people who already face major inequities be further disadvantaged and denied solutions that might improve their quality of life? The need for new service models to be co-created with all stakeholders who use telehealth, including diverse members of the public, has been reinforced in major UK reports [56]. Simple solutions such as having a professional with a client at the user end of telehealth and co-consultation situations would go a long way to improving the experience for both the client and the professional. This, of course, would take funding commitments but would be a small cost to ensure vulnerable rural people facing deprivation have the same rights of access as urban dwellers.

In the UK, demands for urgent action to address health inequalities and poor health and well-being outcomes in rural and coastal areas are escalating [36], however, calls without concerted action are meaningless. Given the significant rural geography and millions of UK residents that reside outside urban cities, rural UK research and dissemination of learnings that demonstrate tangible and measurable health and wellbeing outcomes must be an urgent priority. This study makes a small contribution to embryonic rural UK research.

### Limitations

While the questionnaire was developed from key published literature and met the aims of this study, pilot testing only occurred with four members of the IAPT team. Further testing of the validity of the questionnaire is recommended for further research. There are inherent limitations in self-reported questionnaires, particularly related to social desirability bias. There is a risk that participants in this study felt compelled to answer positively, however, the results indicate that participants perceived benefits and barriers.

Data in this study were collected over a three-month period (April-June 2021) which provides an understanding of how practitioners felt after one year of working with telehealth approaches rather than more immediate responses. Staff may have adjusted to delivering IAPT services via telehealth which could explain the high levels of satisfaction reported in this survey.

Future studies should collect data from users of IAPT via telehealth as well as those who have had difficulty or challenges engaging with telehealth. The cross-sectional descriptive design of this study limits the extent to which the findings are generalisable to IAPT services elsewhere and needs to be tested with subsequent data collection in both rural and urban settings.

## Conclusions

While much of the geography in the UK is designated rural, the health and well-being of millions of rural UK residents has attracted limited interest. The idyllic representation of rural UK hides substantial pockets of deprivation, major issues regarding healthcare accessibility, and poor health and well-being outcomes. Telehealth has been promoted as a panacea for healthcare accessibility in rural communities, but a myriad of barriers has impacted timely uptake. This study makes a significant contribution to both rural and telehealth literature through descriptions of clinician’s experiences in delivering the world’s largest publicly funded, evidence-based psychological program in a rural country in the United Kingdom. The COVID-19 pandemic created a massive shift in the global uptake of telehealth, with this study demonstrating improved access for rural people. While telehealth has captured the attention of policymakers and practitioners internationally, there is a risk that going forward the focus of technological development will centre on the needs of urban centres. The need for fit-for-purpose infrastructure and resourcing, education and training, and support for those with complex needs living in rural communities must not be compromised through urban paternalism and policy and practice driven from an urban stance.

## Data Availability

The datasets used during the current study are available from the corresponding author on reasonable request.

## Abbreviations

CBT: Cognitive behavioural therapy
cCBT: Computerised cognitive behavioural therapy
IAPT: Improving Access to Psychological Therapies
MOOCs: Massive Open Online Courses
NHS: National Health Service
NICE: National Institute for Health and Care Excellence
UK: United Kingdom
US: United States
